# Advancing Cardiomyocyte Maturation: Current Strategies and Promising Conductive Polymer‐Based Approaches

**DOI:** 10.1002/adhm.202303288

**Published:** 2024-02-20

**Authors:** Kamil Elkhoury, Sacha Kodeih, Eduardo Enciso‐Martínez, Ali Maziz, Christian Bergaud

**Affiliations:** ^1^ LAAS‐CNRS, Université de Toulouse, CNRS Toulouse F‐31400 France; ^2^ Faculty of Medicine and Medical Sciences University of Balamand Tripoli P.O. Box 100 Lebanon; ^3^ School of Engineering and Sciences Tecnológico de Monterrey Nuevo León 64849 México

**Keywords:** cardiomyocyte maturation, cardiovascular research, conductive polymers, human induced pluripotent stem cells, physiological stimuli

## Abstract

Cardiovascular diseases are a leading cause of mortality and pose a significant burden on healthcare systems worldwide. Despite remarkable progress in medical research, the development of effective cardiovascular drugs has been hindered by high failure rates and escalating costs. One contributing factor is the limited availability of mature cardiomyocytes (CMs) for accurate disease modeling and drug screening. Human induced pluripotent stem cell‐derived CMs offer a promising source of CMs; however, their immature phenotype presents challenges in translational applications. This review focuses on the road to achieving mature CMs by summarizing the major differences between immature and mature CMs, discussing the importance of adult‐like CMs for drug discovery, highlighting the limitations of current strategies, and exploring potential solutions using electro‐mechano active polymer‐based scaffolds based on conductive polymers. However, critical considerations such as the trade‐off between 3D systems and nutrient exchange, biocompatibility, degradation, cell adhesion, longevity, and integration into wider systems must be carefully evaluated. Continued advancements in these areas will contribute to a better understanding of cardiac diseases, improved drug discovery, and the development of personalized treatment strategies for patients with cardiovascular disorders.

## Graphical abstract

Cardiovascular diseases impose a substantial global burden, underscoring the necessity for effective drug development strategies reliant on the maturation of cardiomyocytes. This review explores the potential of cardiomyocytes derived from human induced pluripotent stem cells, focusing on strategies for achieving adult‐like maturity. Innovative electro‐mechano‐active polymer‐based scaffolds (EMAPS), based on conductive polymers, emerge as a promising platform in this highly urgent quest.

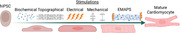

## 1 Introduction

Cardiovascular diseases (CVDs) are a leading cause of death worldwide, accounting for ≈17.9 million deaths each year, which represents 31% of all global deaths.^[^ ^1^, ^2^ ^]^ In addition to the human toll, CVDs also impose a significant economic burden. The economic burden of CVD was estimated to be €210 billion per year by the European Heart Network in 2015, which remains the most recent estimate of the costs of CVD in EU countries.^[^ ^3^ ^]^ In the United States, the American Heart Association estimated that the total cost of CVD is expected to increase from USD 555 billion per year in 2015 to a staggering USD 1.1 trillion per year by 2035.^[^ ^4^ ^]^ In Canada, around CAD 22 billion per year is the estimated cost of CVD.^[^ ^5^ ^]^ In Australia, it is estimated that, between 2020 and 2029, the costs of CVD will exceed AUD 61.89 billion for direct healthcare costs and AUD 78.75 billion in the form of indirect costs, such as the loss of productivity.^[^ ^6^ ^]^ Given the high prevalence and economic impact of CVDs, there is a critical need for innovative approaches to prevent, diagnose, and treat these conditions.

Despite the critical need for new and transformative treatments to alleviate heart muscle cell death and dysfunction, only four new drugs that target cardiac muscle have been approved by the US Food and Drug Administration between 2011 and 2019, compared to 90 novel drugs for cancer.^[^ ^7^ ^]^ 54% of novel therapeutics fail in the most expensive phase 3 of clinical trials, with 57% of those failures due to inadequate efficacy.^[^ ^8^ ^]^ While animal studies often show promise, many drugs fail to demonstrate anticipated efficacy during human clinical trials due to significant physiological differences in organ anatomy, disease progression, and drug metabolism. For example, the human heart beats at a rate of 60 BPM, weighs 300 grams, and has large atria and a long refractory period, whereas the mouse heart beats at a faster rate of 500–600 BPM, weighs only 0.2 grams, and has smaller atria and a short refractory period.^[^ ^9^ ^]^ Added to genetical differences, these physiological differences have led to a lack of new drug candidates being put forward for development, which has resulted in a dry pipeline for cardiac drugs.

The use of adult cardiomyocytes (CMs) in vitro in the development of cardiac disease treatments can increase the approval rate of drugs by providing more accurate and representative models of human cardiac function.^[^ ^10^ ^]^ Moreover, human induced pluripotent stem cells (hiPSCs) are a promising tool for generating human cardiac cells for drug discovery, disease modeling, and regenerative therapies.^[^ ^11^ ^]^ Significant progress has been made in the field of hiPSCs and cardiac differentiation over recent years, showcasing substantial progress in differentiation protocols, cryopreservation strategies, and the scale‐up of hiPSC‐derived CMs (hiPSC‐CMs). Most hiPSC‐CMs differentiation protocols use small molecules on 2D chemically coated surfaces, with a notable but limited adoption of commercially available hiPSC‐CMs (probably due to their higher cost and chemically undefined nature).^[^ ^12^ ^]^ Key considerations of the cryopreservation of hiPSC‐CMs include the optimal differentiation day for freezing, the use of pro‐survival treatments, and factors such as cell density, solution volume, freezing rates, and storage conditions.^[^ ^13^ ^]^ Despite variability in methodologies, cryopreservation has been shown to yield high viability, recovery, and purity (>90%),^[^ ^14^ ^]^ which is crucial for the hiPSC‐CMs' long‐term storage and supports biobanking initiatives. Scaling up the production of hiPSC‐CMs requires the replacement of traditional small‐scale culture systems, which are limited in their cell production yield and scalability, with perfusion, rotating‐wall, or spinner‐flask bioreactors that offer increased productivity and optimized culture conditions.^[^ ^15^ ^]^

Despite these significant advancements, hiPSC‐CMs often exhibit an immature phenotype that limits their potential applications.^[^ ^11^ ^]^ Artificial tissue scaffolds that replicate the natural 3D environment can be engineered to drive the immature hiPSC‐CMs toward the adult‐like state. However, the big challenge relies upon engineering these artificial scaffolds to be able to mimic the architecture, biochemical, and electromechanical of the cardiac microenvironment.^[^ ^16^, ^17^ ^]^ In the native heart, cardiac cells are highly aligned in a specific orientation that allows for efficient contraction and relaxation of the heart.^[^ ^18^ ^]^ This alignment is not only important for the propagation of electrical impulses through the heart, but for the efficient mechanical function of the heart as well.^[^ ^19^ ^]^ Moreover, systemic and local biochemical cues are essential for the proper maturation of functional cardiac cells as any disruptions or imbalances in these cues can lead to developmental defects.^[^ ^20^ ^]^ In addition, studies have demonstrated that mechanical loading and electrical stimulation (E‐stimulation) can enhance the maturation of CMs by positively affecting their gene expression, force generation, and calcium handling.^[^ ^21^, ^22^, ^23^ ^]^

Electrically conductive materials, such as gold nanorods,^[^ ^24^ ^]^ graphene,^[^ ^25^ ^]^ MXene nanoparticles,^[^ ^26^ ^]^ and carbon nanotubes (CNTs),^[^ ^27^ ^]^ are widely used in cardiac tissue engineering, where scaffolds require good electrical conductivity to effectively propagate electrical impulses.^[^ ^28^ ^]^ Conductive polymers (CPs) are one type of electrically conductive materials that hold great promise for cardiac maturation as they offer unique advantages in biomedical applications.^[^ ^29^, ^30^ ^]^ CPs can be used to create fiber‐like scaffolds with aligned structures, which is beneficial for the differentiation and maturation of CMs, as it mimics the native cardiac tissue's organization.^[^ ^19^, ^31^ ^]^ Their intrinsic electrical conductivity allows for the delivery of electrical cues to the seeded cells, promoting the development of organized sarcomeres and enhancing contractility and beating synchronization. Moreover, CPs enable the achievement of E‐ and electromechanical (EM‐) stimulations, further driving the maturation process of CMs. The integration of CPs into tissue engineering approaches provides a biomimetic environment for CMs, enhancing their functionality and potential applications in drug screening, disease modeling, and regenerative medicine for cardiovascular disorders.^[^ ^32^ ^]^

In this review, a comparative analysis is first presented between hiPSC‐CMs and the four distinct stages of CM development, highlighting the importance of achieving mature CMs for disease modeling and therapeutic applications. The challenges in maturing hiPSC‐CMs are explored, and various strategies such as topographical, biochemical, mechanical (M‐), E‐, and EM‐stimulations are discussed to promote their functional development. Furthermore, an in‐depth analysis is presented on how CPs can provide a promising platform for hiPSC‐CMs maturation and how the integration of CPs into tissue engineering approaches provides a biomimetic environment for CMs, making them a valuable tool in advancing the field of cardiac tissue engineering. These discussions provide crucial insights into the current state‐of‐the‐art of hiPSC‐CMs maturation and their potential for disease modeling, drug discovery, and regenerative therapies.

## 2 Comparison of hiPSCs‐CMs and the Four CMs’ Developmental Stages

Although there have been significant advancements in the creation of differentiation protocols, the immature nature of hiPSC‐CMs may still pose a challenge for modeling genetic cardiac diseases that typically present in adulthood. This section outlines maturational differences between the hiPSC‐CMs and the four CMs developmental stages which consist of early fetal, late fetal, neonatal, and adult stages.

### 2.1 Morphology

**Figure 1:**
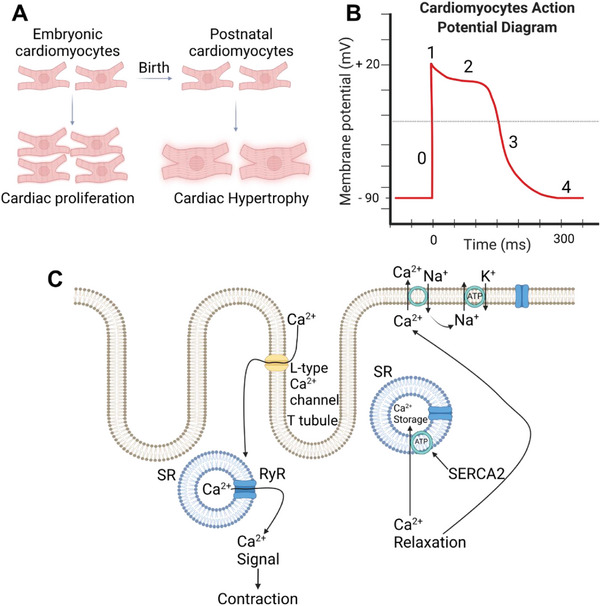
A) Morphological differences in proliferating and hypertrophied cardiac cells. B) Action potential curve of cardiac cells. C) Excitation‐contraction coupling and relaxation in cardiac muscle. Created with BioRender.com.

Cardiac cell development falls under two stages. The first one is called proliferative while still in utero, as embryonic and fetal CMs, and the other is hypertrophic (consisting of postnatal cardiac cells).^[^ ^33^ ^]^ The cell division stage is necessary for the proper formation of cardiac structures and mandatory to accommodate the future increase in the cardiac workload whereas the increase of myocardial volume after birth triggers the hypertrophic stage which plays a role in the increase in cardiomyocytic size instead of the number to accommodate this change in pressure (**Figure** **1**A).^[^ ^34^ ^]^

Being rounded, mononucleated, and random, hiPSC‐CMs show the same morphological shape and alignment respectively as the early and late fetal, the prenatal stage. Some at the neonatal stage show a binucleated appearance (not definitive) with an elongated morphology.^[^ ^35^ ^]^ Ahmed et al. stated that, once the CMs reach the adult stage, they become well‐aligned (anisotropic), fully binucleated, and sometimes multinucleated. Characterized by having a well‐developed intracellular compartment comprising the sarcoplasmic reticulum, transverse tubules, and a highly organized arrangement of sarcomeres, both the hiPSC‐CMs and fetal CMs lack this well‐formed and extensive organization.^[^ ^36^ ^]^ Karbassi et al. stated that immature CMs show characteristically the same rod‐like morphological structure as the adult CMs without clear myofibril alignment. In the process of developing CMs, a unity between adjacent CMs happens forming a way of communication with mature mechanical and electrical junctions, forming a functional syncytium (**Table** **1**).^[^ ^21^ ^]^

**Table 1:** Summary of the nine maturational differences between the hiPSC‐CMs and the four CMs’ developmental stages.

		Early fetal CMs	Late fetal CMs	Neonatal CMs	Adult CMs	hiPSC‐CMs	Ref.
Morphology	Shape	Round	Round	Elongated	Elongated	Round	[35]
	Surface area	1000–1300 µm^2^	1000–1300 µm^2^	<<1000–>10 000 µm^2^	10 00–14 000 µm^2^	1000–1300 µm^2^	
	Alignment	Random	More than early fetal CMs	More than late fetal CMs	Anisotropic	Random	
Contractility	Sarcomere organization	Z disks	Z disks, I bands, A bands, M lines	Z disks, I bands, A bands, M lines, H zones	Z disks, I bands, A bands, M lines, H zones	Z disks and sometimes I bands	[38, 39, 40, 41, 42]
	Force	0.4 mN mm^−2^	0.4 mN mm^−2^	0.8–1.2 mN mm^−2^	10–50 mN mm^−2^	0.1–0.5 mN mm^−2^	
	Proteins	α‐MHC > β‐MHC MLC2a and MLC2v cTnT1 and cTnT2 Slow skeletal troponin I	α‐MHC > β‐MHC MLC2a and MLC2v cTnT1 and cTnT2 Slow skeletal troponin I	β‐MHC > α‐MHC MLC2v cTnT3 and cTnT4 Slow skeletal troponin I	β‐MHC > α‐MHC MLC2v cTnT3 and cTnT4 Cardiac troponin I	Varying amounts of MHC MLC2a and MLC2v Low levels of expression of troponin isoforms	
Electro‐physiology	Resting membrane potential	−40 mV	<−40 mV	<<−40 mV	Stable −85 mV	Unstable −50 to −60 mV	[35, 44]
	Contract	Asynchronous and spontaneous	More synchronous than early fetal CMs	Synchronous with greater electric coupling	Synchronous when stimulated	Asynchronous and spontaneous	
	Calcium channels	High amounts of low T‐type Low amounts of L‐type	Decreased amounts of low T‐type Increased amounts of L‐type	Low T‐type absent Increased amounts of L‐type	Low T‐type absent Increased amounts of L‐type	High amounts of low T‐type Low amounts of L‐type	
Calcium handling		No transverse tubules	Only small indentations of the sarcolemma	Fully developed transverse tubules after 2–3 weeks	Fully developed transverse tubules	Absence of transverse tubules	[36]
Metabolism	Pathways	Glycolysis	Glycolysis	Oxidative within 7 days	Oxidative	Glycolysis	[48, 52, 53]
	Cristae	Absent	Absent	Developed	Densely packed	Absent	
	Mitochondria	Rounded	Rounded	More ovular shaped	Mature network—ovular shaped—provide ample ATP	Immature	
Gene expression		α‐MHC predominates	α‐MHC predominates	Transition from α‐MHC to β‐MHC	Predominance of β‐MHC, cTnI, and N2B	Predominance of β‐MHC, cTnI, and N2B Low cardiac genes expression	[36, 53, 54]
Proliferation ability		High	Decreased	Active for 7 days then silenced permanently	Zero	Active	[35, 55]
Cell cycle	G1/S phase cyclins level	High	High	Decrease	Low	High	[21]

### 2.2 Contractility

The main contractile apparatus in CMs is called myofibrils. They are specialized cytoskeletal structures showing a striated pattern resulting from their sarcomeres’ arrangement. The thick and thin filaments are in the myofibrils. The thick filaments consist of the myosin and other proteins, the cross bridges are these projections that emanate out of the myosin. The thin filaments comprise the actin filaments, troponin, and tropomyosin.^[^ ^37^ ^]^

The sarcomere in a single skeletal muscle fiber extends from 1 Z line to the other Z line which is a collection of interconnecting cytoskeletal proteins that link the thin filaments that form the I band. At the center of the sarcomere, there is a dark band called the A band. The A band is a zone of overlap between the thick and a part of the thin filaments. At the center of the A band is an H zone comprising only thick filaments. The H band is bisected by an M line which is a group of interconnecting proteins that connect the center of myosin filaments. The I band comprises only thin actin filaments.^[^ ^38^ ^]^

The hiPSC‐CMs show the same distribution as the early fetal stage with only Z disks and sometimes they show some bands. Whereas the late fetal stage shows a Z disk with I and A bands and M lines, the neonatal and adult CM stages show, in addition to the ones in the late fetal, an H zone.

However, all contractile proteins of mature CMs are present in the same or a different isoform in hiPSC‐CMs, sometimes with different levels. For example, the 2 isoforms of the myosin heavy chain, beta‐MHC, and alpha‐MHC, are found in all CM stages and hiPSC‐CMs.^[^ ^39^ ^]^ However, beta‐MHC to alpha‐MHC ratio is lower in both fetal stages (α‐MHC > β‐MHC) than in neonatal and adult CMs stages where β‐MHC > α‐MHC. hiPSC‐CMs show varying amounts of both isoforms depending on culture cells.^[^ ^40^ ^]^

As mentioned previously, hiPSC‐CMs show almost similar characteristics with the early and late fetal stages concerning the myosin light chain protein, both MLC2a and MLC2v are present. After birth, MLC2v takes over in the neonatal and adult CM. This switching occurs when ventricular CMs become mature.^[^ ^35^, ^41^ ^]^

Concerning troponin T molecules, both fetal stages express cTnT1 and cTnT2 which then switch to make cTnT3 and cTnT4 in higher loads. Whereas troponin I is in its skeletal form until reaching the adult stage where it ends up forming cardiac troponin I isoform.^[^ ^42^ ^]^

No troponin expression has been noted in hiPSC‐CMs. Troponin is present on each tropomyosin dimer, and influences its position on the actin filament, and, hence the ability of tropomyosin to inhibit the binding of myosin to the actin filament.^[^ ^43^ ^]^ So, due to this organization, hiPSC‐CMs are not able to contract efficiently. For that, the hiPSC‐CMs contractile forces range from 0.1–0.5 mN mm^−2^ in which the range of both fetal stages falls in (≈0.4 mN mm^−2^). This value increases 2–3‐fold in neonatal CMs to reach a value of 0.8–1.2 mN mm^−2^. Once the CMs reach the adult stage, a huge increase in the contractile force occurs for about 10–50 mN mm^−2^ (Table 1).^[^ ^35^ ^]^

### 2.3 Electrophysiology

Ion channels, gap junctions, and inward and outward movement of ions through the membrane play important roles when addressing the action potential conductions in CMs. The resting membrane potential of cardiac cells starts at −40 mV in early fetal and keeps on declining from one stage to another until reaching a stable value of −85 mV in mature CMs, whereas the hiPSC‐CMs fluctuate unstably between −50 and −60 mV due to the low level of I_K1_ channels.^[^ ^44^ ^]^ Concerning the pacemaker current channels, early fetal CMs show the same distribution as the hiPSC‐CMs with high amounts of channels, their number decreases when going from late fetal CMs until reaching low amounts or absence in mature CMs. The instability in the level of pacemaker channels and the resting membrane potential value contributes to the asynchronous and spontaneous contraction of hiPSC‐CMs, the same for the early fetal CMs.^[^ ^45^ ^]^ the contraction becomes more and more synchronized with an electrical coupling in neonatal CMs.^[^ ^35^ ^]^ Adult CMs only beat when stimulated with a conduction velocity of ≈60 cm^−1^ s and an upstroke velocity around 150–350 V s^−1^ compared to 10‐–20 cm^−1^ s and 10–50 V s^−1^ respectively for hiPSC‐CMs which also shows 6–50‐folds lower than the Vmax of the adult CMs.^[^ ^46^ ^]^ CMs express two different types of calcium channels that differ in their distribution from CMs to another, in addition to the electrochemical characteristic differences. Those 2 types are the T‐type and the L‐type calcium channels. The low T‐type calcium channels are highly abundant in early fetal CMs and hiPSC‐CMs,^[^ ^45^ ^]^ decrease in late fetal and become completely absent in neonatal and adult CMs. Instead, the L‐type calcium channels are present in low amounts in early fetal CMs, and hiPSC‐CMs then progressively increase in the late fetal, neonatal, and adult CMs.^[^ ^35^ ^]^

The action potential curve of a cardiac cell shows ≈110 millivolts spike amplitude, starting with ≈−90 mV to a value of ≈+20 mV. AP in cardiac cells can be divided into four phases. Phase 0, also called the depolarization phase, is where the stimulation of cardiac cells leads the voltage‐gated sodium channels to open. An inward sodium flux renders the potential more positive of ≈+20 mV just before Na^+^ channels close. The initial repolarization or phase 1, occurs after the Na^+^ channels close and the K^+^ channels open marking slight repolarization until reaching the plateau or phase 2, where Ca^2+^ channels open and the fast K^+^ channels close, the combination of increasing the influx of calcium and decreasing potassium efflux. Phase 3, rapid repolarization occurs when calcium channels close and the slow K^+^ channels open permitting potassium ions rapid efflux, ends the plateau returning the cell membrane potential to its resting state, Phase 4, also called the resting membrane potential of −85 to −90 mV (Figure 1B).^[^ ^47^ ^]^ hiPSC‐CMs and adult CMs show approximately the same range of action potential amplitude compared to fetal CMs which is 3–6‐fold lower.^[^ ^48^ ^]^

### 2.4 Calcium Handling

The excitation‐contraction coupling (ECC) of CMs is mainly mediated by calcium (Ca^2+^). For a contraction to occur, the action potential is transmitted to the transverse tubules (T‐tubules), which are internal extensions of the cell membrane, and the sarcoplasmic reticulum system.^[^ ^49^ ^]^ Calcium is released when the AP reaches the T tubules, voltage changes, and this change is detected by dihydropyridine receptors linked to a calcium channel called ryanodine receptor channels (RyR receptors).^[^ ^50^ ^]^ Their activation triggers calcium channels to open and release Ca^2+^ in the sarcoplasm, which stays for milliseconds, causing muscle contraction by binding to troponin C and causing the sliding of the myofilament. However, a continuous active calcium pump called sarcoplasmic/endoplasmic reticulum calcium ATPase 2a (SERCA2a) uptake the calcium back to the sarcoplasmic tubules, by the sarcoplasmic reticulum Ca^2+^ ‐ATPase (SERCA). Also, a protein called calsequestrin present in the reticulum is capable of binding a high number of calcium molecules (Figure 1C).^[^ ^51^ ^]^

The early fetal CMs and the hiPSC‐CMs have no T‐tubules, the late fetal shows only small indentations of the sarcolemma, and the neonatal and adult CMs develop full transverse tubules. In adult CMs, well‐developed T‐tubules and SR regulate Ca release (CICR) and fast ECC whereas, L‐type channels regulate CICR and slow ECC in hiPSC‐CMs.^[^ ^36^ ^]^

### 2.5 Metabolism

Mitochondrial maturation is not just a byproduct of CMs maturation, it is the driving force behind the maturation of CMs. Mature CMs are characterized by an ovular shape, and densely packed cristae, occupying 35% of the cell volume with intermyofibrillar, subsarcolemmal, and perinuclear organization. In contrast, early and late fetal CM's mitochondria are smaller in size and rounded, with no clear sarcomeric localization and absence of well‐defined cristae.^[^ ^52^ ^]^ However, mitochondria in neonatal CMs become more ovular‐shaped with an increase in amount and size and well‐developed cristae. The mitochondria of fetal CMs and hiPSC‐CMs express similar characteristics with perinuclear disorganization.^[^ ^35^ ^]^

During gestation, the main source of nutrients is the mother‐fetus glucose gradient, and the fetus receives nutrition that is low in fatty acids from the placenta, which is why both early and late fetal CMs rely on glycolysis as a major source of energy production. In contrast, breastmilk has a high fatty acid content, neonatal CMs switch to an oxidative metabolism to meet the cell's demand and stay in mature CMs. Same as fetal CMs, hiPSC‐CMs rely also on glycolysis.^[^ ^48^, ^53^ ^]^

### 2.6 Gene Expression

CM maturation, proliferation, and the underlying characteristics of each stage are dictated by the gene expression at a time. Genes undergo a process called isoform switching while transitioning from one CM stage to another. Cui et al. show that the expression pattern of these maturation‐related genes is almost similar in mice and humans.^[^ ^56^ ^]^ For example, there is an overall increase in mature sarcomere components, transitioning from fetal to adult period.^[^ ^37^ ^]^ Cardiac myosin heavy chain MHC (also known as MYH) undergoes switching from alpha‐MHC (MYH6) to beta‐MHC (MYH7) where the beta form is predominant in adult CM. Kreipke et al. noted that this transition, in opposition to the human myosin gene, occurs from beta–MHC to alpha–MHC in mouse CMs.^[^ ^53^ ^]^ Three Troponin I (TnI) genes, TNNI1, TNNI2, and TNNI3 each coding different TnI: slow skeletal (ssTnI), fast skeletal (fsTnI), and cardiac (cTnI) respectively.^[^ ^54^ ^]^ The slow skeletal characterize the hiPSC‐CMs then the cTnI predominates in adult CMs. As such, Titin (TTN) also has three isoforms, N2B, N2BA, and FCT (fetal cardiac titin). The predominant gene isoform in hiPSC‐CMs is N2BA switching to N2B in adult CMs (TTN‐N2BA to TTN‐N2B).^[^ ^36^ ^]^ Moreover, electrophysiologically, adult CMs show an increase in Ca^2+^ handling molecules like SERCA2 (sarcoplasmic reticulum ATPase), CAV3 (caveolin 3), also ventricular ion channels, such as KCNH2 (potassium voltage‐gated). However, they show a decrease of automaticity ion channels (decrease HCN4 channel expression). In addition, the maturation of CMs is driven by the downregulation of glycolytic genes, and the upregulation of genes involved in mitochondrial biogenesis, oxidative phosphorylation, and fatty acid metabolism.^[^ ^37^ ^]^

### 2.7 Proliferation Ability

The heart as a terminally differentiated organ, in adult stages, is vulnerable to losing its function, without any regenerative capabilities. The inability of the remaining CMs to regenerate decreases the cardiac cells’ normal functioning leading to ischemia.^[^ ^57^ ^]^ On the contrary, early and late fetal CMs show a high regenerative capacity that decreases when passing from one stage to another. Like Early Fetal CMs, hiPSC‐CMs have the highest proliferation abilities.^[^ ^35^ ^]^ Postnatally, Zhu et al. noticed that myocyte proliferation led to the presence of a strong recovery trend in neonatal porcine hearts only in the first 2 days following an acute myocardial infarction.^[^ ^55^ ^]^ Studies have shown that creating a gradual systemic hypoxaemic environment up to 1 week, in mice adult CMs, leads to a modest self‐renewal ability.^[^ ^58^ ^]^ Near the injured CMs cells, an increase in enzymes responsible for glycolysis and pyruvate metabolism causes an increased ability of the CMs to regenerate after injury.^[^ ^59^ ^]^ Also, Wu et al. found that CM‐specific deletion of the LRP6 gene in knockout mice increased the proliferation of CMs at different developmental stages including the pluripotent stem cell‐derived CM.^[^ ^60^ ^]^ However, the reason why adult CMs have limited proliferative capacities is due to their inability to enter the cell cycle again.^[^ ^61^ ^]^

### 2.8 Cell Cycle

**Figure 2:**
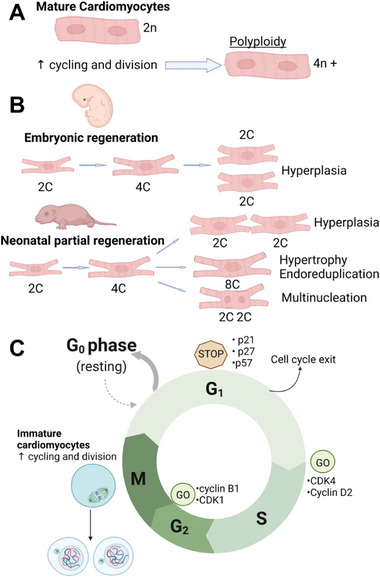
A) CMs undergoing polyploidy. B) The hypertrophy, multinucleation, and endoreduplication of neonatal CMs in comparison to the embryonic regeneration process in mice. C) Cell cycle regulation by transcription factors, cyclins, cyclin‐dependent kinases (CDKs), and CDK‐ inhibitors CKIs. Created with BioRender.com.

CVDs are still a major cause of death worldwide. The poor cardiovascular outcome is denoted by a limited regeneration of adult CMs after injury. While some of the struggles are attributed to the postmitotic adult CMs.^[^ ^62^ ^]^ Nonetheless, neonatal CMs exhibit a higher level of regenerative capacity.^[^ ^63^ ^]^ That will cease shortly after birth, day 7, after transitioning from a hypoxic to an oxygen‐rich environment postnatally, coinciding with nuclear binucleation and cell cycle arrest.^[^ ^64^ ^]^ All future CM growth occurs by hypertrophy,^[^ ^65^ ^]^ after undergoing cell cycle variant under polyploidy either by DNA synthesis and nuclear division without cytokinesis (also called endoreduplication) or without nuclear division, cytokinetic mitosis, to make polyploid nuclei (**Figure** **2**A). This polyploidy, with a range of 4 n to 64 n, increases with age in humans, for example, and occurs while going from ectothermy to endothermy leading to CM cell cycle exit (Figure 2B).^[^ ^21^, ^66^ ^]^

The cell cycle is controlled by many molecules like transcription factors, cyclins, cyclin‐dependent kinases (CDKs), CDK‐inhibitors CKIs (like INK4 and CIP/KIP), and microRNA molecules. Both cyclins and CDKs are positive modulators of the cell cycle and increase during mitosis. Cyclin Ds subtypes enhance the cell cycle entry and have an affinity to CDK4 (Figure 2C).^[^ ^67^ ^]^ Karbassi et al. showed that the level of mRNA coding for G1/S phase cyclins is high throughout all stages, but their protein levels decrease when going from fetal to adult CMs state. On the other hand, CDKs are formed during embryonic heart development and stay stable in neonates, but their level of expression in adult CMs decreases. CKIs like p21, p27, and p57, show increased levels in fetal and neonatal CMs, except for p57 which decreases and is important in cell cycle exit. As such, the level of cyclins and CDKs decreases in hiPSC‐CMs.^[^ ^21^ ^]^ β‐catenin and Yes‐associated protein 1 (YAP1) dependent signaling, are 2 major proteins when suppressed cause hiPSC‐CMs cell cycle arrest.^[^ ^68^ ^]^ Also, Mahmoud et al. demonstrated that the deletion of the Meis1 gene in adult CMs using knockout mice induces cell cycle re‐entry.^[^ ^69^ ^]^

### 2.9 hiPSC‐CMs Maturity Gap

It is important to note that the visualization of the maturation of hiPSC‐CMs as a single phenomenon is not accurate, as it is a complex phenomenon that is controlled by multiple signaling networks.^[^ ^70^ ^]^ To enhance the maturity of the hiPSC‐CMs to adult‐like CMs, several methods are used, such as metabolic and hormonal interventions, and chemical, topographical, M‐, E‐, and EM‐ stimulations.^[^ ^36^, ^71^ ^]^ Some hormonal interventions like using neuregulin 1β (NRG1) in combination with insulin‐like growth factor 1 (IFG1) have been shown to enhance the force‐frequency produced, but it turns out that the total force production was reduced.^[^ ^48^ ^]^

Also, the expression of the I_k1_ channels (inward‐rectifier potassium channel) coded by the KCNJ2 genes shows lower levels in hiPSC‐CMs compared to the late fetal stages.^[^ ^35^, ^45^ ^]^ Karbassi et al. showed that adult CMs undergo atrophy, dedifferentiate, or die after placement in vitro cultures.^[^ ^21^ ^]^ However, fully developed transverse tubules are completely formed in mature native CMs while cultured in vitro, in opposition to the hiPSC‐CMs.^[^ ^35^ ^]^ In addition, the morphological difference between adult and immature CMs limits the hiPSC‐CMs' normal development to ensure the same conditions as the desired regenerative therapy.^[^ ^21^ ^]^ Once the conditions of the fetal stages of CM development are all well known, the hiPSC‐CMs can then progress and resemble the neonatal CMs stage and thus the adult CMs.

## 3 Closing the Gap in hiPSC‐CMs Maturity

Stem cell differentiation technologies with the aim of creating functional scaffolds to regenerate the myocardium as an alternative to organ transplant are being tested. Multiple efforts include the use of bone marrow and endogenous cardiac stem cells, but the low yield and differentiation efficiency remain a challenge for clinical translation.^[^ ^72^, ^73^, ^74^ ^]^ Efforts to recapitulate adult‐like CMs are being developed. For instance, Kadota and colleagues transplanted hiPSC‐derived cardiac progenitor cells and hiPSC‐derived CMs in neonatal rats to study in vivo maturation, however cells partially matured after 3 months.^[^ ^75^ ^]^ This evidence strongly suggests the importance of novel hiPSC‐CM differentiation strategies to recapitulate adult‐like phenotype cardiac myocytes.

Early maturation protocols tested the potential of hiPSC‐CMs long‐time culture mimicking the in vivo development of adult CMs. Kamakura and colleagues tested this approach culturing hiPSC‐CMs embryoid bodies for 14, 180, and 360 days finding unaligned and immature myofibrils on day 14, tightly packed myofibrils, mature Z‐, A‐, H‐ and I‐ bands on day 180 and mature M‐ bands only in day 360.^[^ ^76^ ^]^ To acquire additional mature markers such as ECC, enhanced calcium handling, T‐tubule formation, and ECM deposition scientists have explored other maturation methodologies.

Engineered heart tissues (EHTs) are being developed for disease modeling, preclinical drug testing, and cardiovascular research applications. The potent capability to genetically modify patient‐specific hiPSC‐CMs renders them a robust technology for investigating inherited cardiomyopathies,^[^ ^77^, ^78^, ^79^, ^80^, ^81^, ^82^, ^83^, ^84^, ^85^ ^]^ cardiac fibrosis,^[^ ^86^, ^87^, ^88^, ^89^ ^]^ and ischemia‐reperfusion injuries (IRI),^[^ ^90^, ^91^, ^92^, ^93^, ^94^ ^]^ with the significant advantage of ensuring the relevance of studied mutations to humans, distinguishing them from traditional animal models.^[^ ^95^ ^]^ Cohn et al. utilized CRISPR to engineer four hiPSC‐CMs models of hypertrophic cardiomyopathy (HCM) and identified the p53 pathway as a potential therapeutic target candidate for HCM patients.^[^ ^77^ ^]^ Dai et al. utilized patient‐specific hiPSC‐CMs with a dilated cardiomyopathy (DCM) mutation to suggest that addressing sarcomere‐cytoskeleton interactions could offer a promising therapeutic strategy for promoting sarcomere reorganization and contractile recovery in DCM patients.^[^ ^78^ ^]^ Wang et al. produced EHTs using the biowire platform to model cardiac fibrosis, study its pathophysiological features, and screen antifibrotic compounds.^[^ ^86^ ^]^ Using EHTs and a bioreactor, Chen and Vunjak‐Novakovic were able to model IRI, study its key aspects, and reduce its effects through a combination of cardioprotective therapeutics, ischemic preconditioning, and reperfusion conditions modifications.^[^ ^90^ ^]^

Utilizing high‐throughput platforms for preclinical drug screening is essential in minimizing the expenses associated with drug discovery. A novel study by Ribeiro and colleagues introduces a potential platform for high‐throughput screening.^[^ ^96^ ^]^ Their modular platform enables the fabrication of 36 3D EHTs which fit in commercial 12‐well plates. This system allows optical tracking to functionally analyze force, velocity, and time of contraction‐relaxation readouts with tissues under EM‐stimulation. Results demonstrated an enhanced performance in contraction and relaxation parameters of EHT cultured in a maturation medium. In 2013 Agarwal et al. presented a microfluidic heart on a chip with semi‐automated fabrication techniques integrating soft elastomers.^[^ ^97^ ^]^ This study aimed to achieve higher throughput pharmacological studies. Anisotropic cardiac microtissues were engineered on cantilevers of soft elastomers. The cantilever deflection enabled muscular thin films' computation of diastolic and systolic stresses. The microtissues were tested on a reusable fluidic device to assess optical cardiac contractility which includes stimuli such as direct electrical field stimulation in the tissue.

Stromal cells drive functional maturation cues in EHTs. It was shown by Giacomelli et al. that hiPSC‐CMs’ maturation can be enhanced by co‐culturing them with cardiac fibroblasts (CFs) and endothelial cells (ECs) derived from hiPSCs.^[^ ^98^ ^]^ The study demonstrated that this co‐culture improved sarcomeric structures, enhanced contractility, and electrophysiological maturity. This improved maturation was evidenced by enhanced sarcomeric structures, superior contractility, and electrophysiological maturity, facilitated by the formation of connexin 43 gap junctions between hiPSC‐CMs and CFs and the activation of the intracellular cyclic AMP pathway. For co‐culture systems, mixing cell‐type‐specific culture media in ratios corresponding to their respective cell numbers or formulating custom blends with a common base medium enriched by combinations of cell‐type‐specific cytokines and growth factors has been the successful approach for supporting cardiac tissues.^[^ ^1^ ^]^ Nonetheless, the impact of suboptimal media on tissue maturation and function remains not fully understood, constituting a potentially significant yet poorly explored aspect.

Macrophages have been shown to modulate the electrical activity of CMs and support normal atrioventricular nodal conduction by Hulsmans et al.^[^ ^99^ ^]^ Moreover, following a co‐culture of hiPSC‐CMs with different types of macrophages, Long et al. showed that the non‐activated M0 macrophages did not significantly influence the hiPSCs’ cardiomyogenesis, while the pro‐inflammatory M1 macrophages notably reduced both the cardiac differentiation and maturation of hiPSCs. Additionally, although the anti‐inflammatory M2 macrophages did not impact the CMs’ yield during the cardiac differentiation, they did play a role in promoting CM maturation.^[^ ^100^ ^]^ This shows that co‐culturing hiPSC‐CMs with immune cells specifically macrophages, governs their proliferation, cardiac differentiation, and maturation.

As previously mentioned, the mature phenotype of adult CMs has several biochemical and biophysical characteristics different from hiPSC‐CMs. It is becoming clear that EM‐stimulation is becoming a major strategy for CM maturation. Novel research is presenting approaches that include platforms with additional stimulation such as biochemical, native architectural features from the ECM, co‐culture methodologies mimicking the cardiac tissue, biomaterial tunability, and emerging biofabrication techniques. Cardiac tissue engineering applications are branching from regenerative medicine to disease modeling and preclinical drug testing. Experts in the field are continuously discovering new types of stimulation and even combining multiple maturation strategies to improve the differentiation of CMs. Among the well‐studied stimuli, the biochemical, topographical, mechanical, and electrical cues govern the most impactful outcomes in the maturation of hiPSC‐CMs.

### 3.1 Biochemical Stimulation

Biochemical cues have been used for years to induce specific cell behavior such as VEGF for vessel sprouting in ECs. In the particular case of hiPSC‐CMs, it has been studied the direct role of thyroid hormone T3 on the performance of potassium channels present in CMs.^[^ ^76^ ^]^ Ulivieri et al. proved that thyroid hormones T3 and T4 regulate the repolarization of the QT‐interval.^[^ ^101^ ^]^ Additionally, glucocorticoids represent a group of hormones that play a key role in myofibril alignment and construction improving the overall maturity of iPSC‐CM.^[^ ^102^ ^]^

The major sources of energy for CM in vivo are glucose and fatty acids. During embryonic and neonatal stages, the mammalian heart heavily relies on aerobic glycolysis for energy generation.^[^ ^103^ ^]^ To avoid this glucose‐dependent pathway and transition CM to adult‐like β‐oxidation of fatty acids in the mitochondria, Correia et al. cultured hiPSC‐CMs in galactose and fatty acid‐containing medium. This approach resulted in improved maturation of hiPSC‐CMs with increased oxidative metabolism, myofibril density, and alignment, improved calcium handling, and contractility.^[^ ^104^ ^]^ After understanding the galactose‐rich medium associated with lipotoxicity, the same group introduced a new method to shift the hiPSC‐CMs metabolism to oxidative pathways. Hu and colleagues used HIF1α‐LDHA small molecule inhibitors which resulted in enhanced sarcomere lengths, mitochondrial number, calcium transient kinetics, and contractile force generation.^[^ ^105^ ^]^ A different study shows the benefit of iPSC‐CM long‐term culture in dextran vinyl sulfone networks functionalized with fibronectin. The DVC matrices enabled increased structural organization of cell‐cell localization and functional calcium flux dynamics.^[^ ^106^ ^]^

Intercellular communication plays a relevant role in CM proliferation, Follistatin‐like 1 (FSTL1) molecule has been shown to increase CM proliferation in animal ischemic models. FSTL1 is secreted by CM and fibroblasts, interplaying a bidirectional communication pathway in the cardiac microenvironment. Peters and colleagues generated an in vitro hypoxia‐damaged cardiac human model to show the FSTL1 positive effect in iPSC‐CM proliferation.^[^ ^107^ ^]^ Taken together the numerous hormones, molecules, and bioactive materials used in the different studies suggest that the biochemical cues are just one of the several key stimuli to induce mature iPSC‐CM.

### 3.2 Topographical Stimulation

The topography of a surface can have a significant impact on the adhesion and differentiation of cells, including CMs. Studies have shown that the topography of a surface can influence the morphology, alignment, and maturation of CMs. The native myocardium is surrounded by three layers of collagen matrix from outer to inner 1) Epimysial fibers coat large CM bundles mechanically stabilizing the tissue 2) Perimysial fibers coat individual CM bundles and finally 3) Endomysial fibers interconnect CM cytoskeletons through costameres.^[^ ^108^, ^109^, ^110^ ^]^ This hierarchical structure creates a unique and vital extracellular matrix topography for CM to grow and contract in a coupled manner.

**Figure 3:**
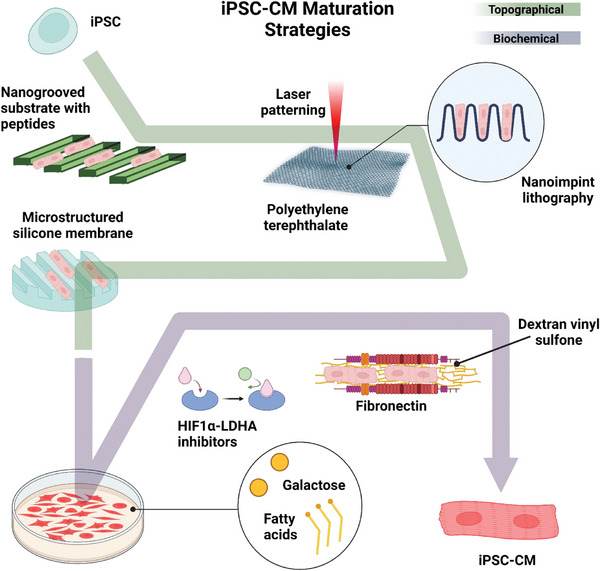
Schematic summary of topographical and biochemical cues for iPSC‐CM maturation. Created with BioRender.com.

**Figure 4:**
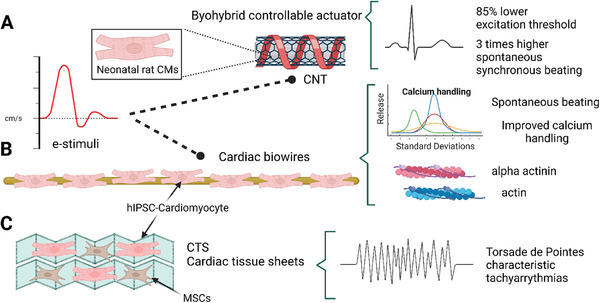
Tissue engineering strategies for cardiac maturation. A) Electrically controllable 3D biohybrid actuator of neonatal rat CMs. B) Cardiac biowire under 3D culture, ECM modification, and E‐stimulation. C) Cardiac tissue sheets resembling torsade de pointes arrhythmias. Created with BioRender.com.

CMs are highly organized cells that are arranged in a specific orientation in native cardiac tissue. The alignment and organization of CMs are critical for the efficient contraction of the heart. In vitro, CMs typically lack this organization, and their maturation can be limited. However, by manipulating the topography of the substrate on which CMs are cultured, it is possible to promote the alignment and maturation of CMs. For example, studies have shown that microgrooved or nanopatterned surfaces can promote the alignment of CMs in vitro, leading to improved sarcomere organization and contractile function.^[^ ^111^, ^112^ ^]^ The topography of the substrate can also influence the expression of genes involved in cardiac development and function, further promoting the maturation of CMs. A research group fabricated nanogrooved substrates with chimeric peptides holding the Arg‐Gly‐Asp (RGD) cell adhesion motif, this topography improved iPSC‐CM organization and structural maturation in grooves within the 700–1000 nm range.^[^ ^113^ ^]^ Cortella et al. introduced a microtopographic dual method of direct laser interference patterning and roll‐to‐roll nanoimprint lithography hot embossing of polyethylene terephthalate as a substrate for iPSC‐CM maturation. This strategy induced morphological changes, anisotropic myofibril alignment, faster calcium reuptake, and positive sarcomeric myofibril assembly.^[^ ^114^ ^]^ Along the topographical stimulation spectra, a research group cultured iPSC‐CM in microstructured silicone membranes which provided a substrate with a native‐like stiffness and structure. The iPSC‐CM acquired rod shape morphology and mature contractile function, calcium handling, and electrophysiology.^[^ ^115^ ^]^ As discussed, multiple biochemical and topographical strategies have been implemented for the enhanced maturation of iPSC‐CM. (**Figure** **3**) A recent strategy used to fabricate cardiac tissue constructs is layering. In 2017, Kawatou et al. mimicked torsade de pointes (TdP) arrhythmias in vitro using 3D cardiac tissue sheets (CTSs) containing a co‐culture of hiPSC‐CMs and non‐myocytes. TdP characteristic tachyarrhythmias were observed in the CTSs (**Figure** **4**C).^[^ ^116^ ^]^ Although this strategy was used as a disease model, the proposed protocol induced a successful physiologic response which could be translated into cardiac maturation research.

### 3.3 E‐Stimulation

In addition to mechanical stimulus as a maturation strategy, E‐stimulation has relevant physiological effects as well. Recently developed biomaterials integrate electrically conductive properties as electroactive biomaterials can transmit E‐, electrochemical, and EM‐stimulations to the cells.^[^ ^117^ ^]^ The robust possibilities of E‐stimulation technology have enabled the use of carbon‐based nanomaterials, gold nanomaterials, and electric CPs in cardiac tissue engineering applications. A new technology defined as the biowire is emerging in multiple cardiac regeneration research projects.^[^ ^76^ ^]^ It refers to a developed human cardiac micro‐tissue that resembles physiological characteristics such as micro‐architecture and electrical stimuli to provide improved hiPSC‐CMs maturation.

Nunes et al. developed a cardiac biowire consisting of compacted PSC‐CMs seeded on a collagen/Matrigel matrix around anchored sutures E‐stimulated gradually from 0 to 3 Hz and from 0 to 6 Hz for 7 days.^[^ ^76^ ^]^ The second group resulted in increased formation of intercalated discs and mitochondria, improved sarcomere organization with the presence of H zones, I bands, and Z disks as well as higher cellular surface area, conduction velocity, and synchronous beating.

The use of multi‐walled CNTs has been implemented in cardiac cell maturation research. A study led by Elkhenany and colleagues investigated cardiac cell behavior with E‐stimulation in electrically conductive GelMA scaffolds integrated with 2 and 5 nm diameter multi‐walled CNTs. After exposition to a 5 V, 1 Hz, and 50 ms pulse width an overexpression of sarcomeric α‐actinin and connexin 43 was observed.^[^ ^118^ ^]^ Xiao et al. microfabricated a platform to mimic the native cardiac bundle which they named perfusable cardiac biowire. This bioreactor was seeded with neonatal rat CMs and was E‐stimulated with the field parallel to the biowire long axis to assess the maturation effect on the cells. Results showed higher cTnT positive structures compared to disorganized structures in control groups, moreover, stronger connexin 43 expression and better coupling between CMs were observed. It was noted that stimulated biowires improved their mechanical properties with increased stiffness and Young modulus.^[^ ^119^ ^]^

A posterior approach to the cardiac biowire developed by Nunes et al. was carried out by Sun and Nunes in 2016. Here a multi‐stimuli platform was engineered including 3D culture, ECM modification, and E‐stimulation. hiPSC‐CMs were cultured for 2 weeks and demonstrated upregulated sarcomeric α‐actinin and actin expression, improved calcium handling, and spontaneous beating activity (Figure 4B).^[^ ^120^ ^]^ Shin et al. developed an electrically controllable 3D biohybrid actuator for neonatal rat CMs. This system was assembled in scaffolds containing hydrogel GelMA plates embedded with 50–100 nm diameter CNTs microelectrode arrays. The E‐stimulation improved maturation cues: three times as much spontaneous synchronous beating rates and 85% lower excitation threshold in contrast to control cell culture in GelMA hydrogels (Figure 4A).^[^ ^27^ ^]^

### 3.4 M‐Stimulation

Evidence indicates that in vitro cultured primary CMs in the absence of mechanical loading generate sarcomere disassembly and weak contractile function.^[^ ^121^ ^]^ In line with the relevance of M‐stimulation for cardiac regeneration, numerous scientists report advances in this area. Nguyen's group designed a microfluidic device with cultured chick embryonic CMs subjected to multi‐M‐stimulation via cyclic fluid flow, chamber pressure, and strain achieving proper calcium handling, increased cardiac troponin, and SERCA expression in contrast to control.^[^ ^122^ ^]^ Another approach that enabled endothelial network formation consists of a perfusable bioreactor containing EHT subjected to 5% stretch and 1 Hz frequency resulting in enhanced cardiac troponin, connexin 43, and MHC expression.^[^ ^123^ ^]^

EHTs are 3D platforms with seeded CM that enable biophysical stimulation. Major remarks on M‐stimulation report hiPSC‐derived EHT with CM hypertrophy and alignment along with the Frank‐Starling relationship.^[^ ^124^ ^]^ Moreover, EHT with hiPSC‐CMs and stromal cells under cyclic stretch in contrast to static stretch increases force production and sarcomere length.^[^ ^125^ ^]^

Mihic's group investigated the behavior of an EHT after in vivo implantation on ischemic rat hearts. hESC‐derived CMs were seeded into a gelatin sponge and subjected to uniaxial cyclic stretching at 12% elongation and 1 Hz frequency on a 3‐day period. The outcome showed effective electrical coupling, highly organized Z disks as well as upregulated KCNJ2 ion channels and connexin 43 expression.^[^ ^126^ ^]^ Ronaldson‐Bouchard et al. developed a collagen‐based EHT with hiPSC‐CMs with auxotonic mechanical conditioning and achieved diad T‐tubule system formation, enhanced β‐adrenergic inotropic response, elongated myofibres, and high twitch‐to‐resting tension.^[^ ^127^ ^]^ Rogers and colleagues created a comprehensive cardiac ventricular stretching emulation system with included the contraction, ejection, and relaxation phases of the cardiac cycle. A system with a collagen/Matrigel‐coated PDMS membrane platform containing hiPSC‐CMs was perfused by a pressure gradient‐powered pump. It was concluded that physiological loading implementation in a gradual manner is vital for increased cell viability.^[^ ^128^ ^]^

### 3.5 EM‐Stimulation

As previously mentioned, the electrical and mechanical forms of stimulation create positive outcomes in the stem cell maturation efforts to achieve adult‐like CM phenotype. Both stimuli control vital physiological functions including expression of genes encoding myofibril, ion channel, and metabolic proteins.^[^ ^129^ ^]^ An approach to dual stimulation has been addressed by testing static stretch in combination with electrical stimuli of 5 V cm^−1^, 2 Hz, and 5 ms pulse duration on hiPSC‐CMs seeded in a collagen matrix. This approach found enhanced RYR and SERCA expression and contraction force in contrast to single stimuli conditions.^[^ ^22^ ^]^ A previously mentioned study by Ronaldson‐Bouchard et al. also tested a combined stimulus consisting of a ramped E‐stimulation sequence from 2 Hz to 6 Hz for 2 weeks and 2 Hz during one more week along with mechanical loading. This condition generated adult‐sized hiPSC‐CMs and myofibril sarcomere lengths, with a high density of intermyofibrillar mitochondria. Architectural and structural features of the natural myocardium were observed including CM bundles.^[^ ^127^ ^]^

**Figure 5:**
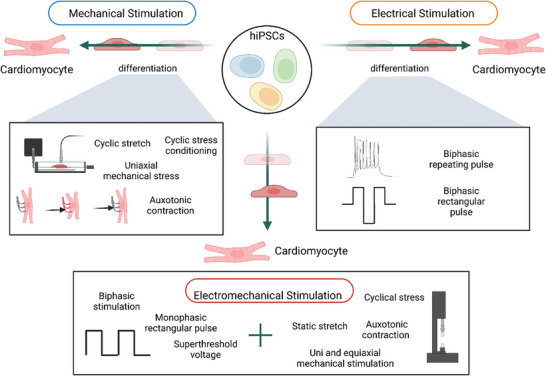
Schematic representation of cardiac regeneration strategies using M‐, E‐, or EM‐stimulations. Created with BioRender.com.

CPs’ use has been shown for EM‐stimulation to drive hiPSC‐CMs differentiation. Gelmi and colleagues developed an EM‐active fiber scaffold composed of an electroactive poly(lactic‐*co*‐glycolic acid) (PLGA) fiber scaffold coated with conductive polypyrrole (PPy) which delivered an EM‐stimulation to hiPSCs.^[^ ^130^ ^]^ This platform was the first to resemble physiological strain to individual cells with cyclic mechanical flow and force. Results exhibit increased expression of cardiac markers and excellent cell viability. In summary, mechanical, electrical, and electromechanical cues are a relevant avenue for hiPSC‐CMs maturation (**Figure** **5**).

### 3.6 Challenges

The complexity of the human CM maturation process is defined by the variety of stimuli and specificity of their timepoint occurrences required for the creation of adult‐like phenotype CMs. Cardiac cells are influenced by biochemical, metabolic, intercellular, and biophysical stimuli. Part of the functionality of adult‐like CMs relies on cellular developmental conditions. For instance, electrical propagation requires cell‐cell electrical coupling via gap junctions. The circumferential distribution of the intercalated disc complex polarizes to the ends of the CM during in vivo maturation. This kind of polarization is inexistent in hiPSC‐CMs standard culture methods.^[^ ^35^, ^129^ ^]^ Scientists working on cardiac maturation strategies face multiple challenges: 1) iPSC‐CM differentiation protocols widely vary, thus the purity and maturation of CMs cannot be compared between studies.^[^ ^131^ ^]^ 2) Scaffold biomaterial composition is mostly different among studies influencing cellular processes.^[^ ^132^ ^]^ 3) Stimuli conditions and approaches change among studies biasing the most successful strategies. 4) Cell lines used for maturation are either neonatal rat CMs, ESC‐CMs, or hiPSC‐CMs limiting the current achievement of reproducibility. 5) There is no standardized guideline to assess the maturation degree of CMs. 6) Terms used to describe the mature phenotype of iPSC‐CM are rather qualitative than quantitative, making the best conditions undetectable. Despite the tremendous progress in cardiac regeneration happening recently and summarized in **Table** **2**, these challenges should be resolved before being able to achieve a successful and reproducible cell maturation into adult‐like CMs.

**Table 2:** E‐ and M‐stimulation approaches to drive the cell maturation into adult‐like CMs.

Study	Cell line	Stimuli			Outcome		Time	Ref.
		E‐stimuli	M‐stimuli	Other	Biochemical	Biophysical		
Trasnplantation of hiPSC‐derived CM to study in vivo maturation in rat neonatal heart	hiPSCs‐derived cardiac progenitor cells hiPSC‐derived CMs	‐	‐	‐	Induction of cardiac troponin I expression	Increased cell size Increased sarcomere length	3 months	[75]
Transplantation of hiPSC‐derived CM to study in vivo maturation in rat adult heart	hiPSCs‐derived cardiac progenitor cells hiPSC‐derived CMs	‐	‐	‐	Increased cardiac troponin I expression	CM hypertrophy	3 months	
Microfluidic device with multi‐M‐stimulation for CM maturation	Chick embryonic CMs	‐	Cyclic M‐stimulation 100 mmHg 15% stretch 2 Hz frequency	‐	Increased calcium‐handling proteins	High beat rate High contractility response to isoproterenol	4 days	[122]
Perfusable bioreactor for EHTs maturation	Neonatal rat heart cells	‐	Cyclic mechanical stretch 5% stretch 1 Hz frequency	Matrix perfusion during M‐ stimulation	Increased cardiac troponin, connexin 43, and MHC expression	Improved contractile function CM alignment along stretch axis	48 h	[123]
Engineering of human myocardium subjected to m‐stimuli	hESC‐derived CMs hiPSC‐derived CMs	‐	Uniaxial mechanical stress	‐	‐	Twofold increase in CM and matrix fiber alignment Enhanced myofibrillogenesis	4 days	[124]
			Cyclic stress conditioning	‐	‐	Increased CM hypertrophy and proliferation rates		
EHT in 3D matrix under M‐stimulation	hESC‐derived CMs	‐	Cyclic stretch 6 mm + 500 um 10% elongation at 1 Hz 5% displacement at 1 Hz	Addition of MSCs	Collagen I, fibronectin, and laminin concentration at cell‐cell junction Z‐discs labeling α‐actinin labeling	CMs interconnected to aligned myofibrils Striated sarcomeric patterning	3 days	[125]
Cyclic stretch effect on hESC‐CMs	hESC‐derived CMs	‐	Uniaxial cyclic stretch 12% elongation 1 Hz frequency	‐	Upregulated KCN2 ion channels Increased connexin 43 expression Increased gap junction expression	Effective electrical coupling Highly organized Z disks	3 days	[126]
Maturation of iPSC‐derived CMs	iPSC‐derived CMs	Monophasic square pulse 3.5–4 V cm^−1^ 2 ms duration	Stretch and auxotonic contraction Ramped M‐stimulation 2–6 Hz	‐	Oxidative metabolism Induced expression of adult‐like: Conduction: ↑ ITPR3,KCNH2↓HCN4 Maturation: ↑ NPPB,MAPK1,PRKACA Ultrastructure: ↑ MYH7,GJA1,TNNI3,AKAP6,GJA5,JPH2 Energetics: ↑ AKAP1,TFAM,PPARGC1A Calcium handling: ↑ CAV3,BIN1,ATP2A2,RYR2,ITPR3	Physiological sarcomere length High mitochondria density (30%) Presence of transverse tubules Elongated myofibres Positive force‐frequency relationship High twitch‐to‐resting tension	4 weeks	[127]
Biowire as a platform for CM maturation	hPSC‐derived CMs	Biphasic repeating pulse 3 V cm^−1^ 1 pulse s^−1^ Pulse duration of 1 ms	‐	‐	Strong expression of a‐actinin and actin Improved calcium handling Enhanced hERG current density Enhanced inward rectifier current density (Ik1)	Cell alignment along axis of suture Improved electrophysiological properties Spontaneous beating activity	2 weeks	[120]
Perfusable cardiac biowire	Neonatal rat CMs	Biphasic rectangular pulse 3.5–4 V cm^−1^ 1.2 Hz frequency 1 ms duration	‐	Peristaltic perfusion of medium and NO	Stronger connexin 43 expression	Higher cTnT positive structures Improved CM coupling Increased stiffness and Young modulus	4 days	[119]
EM‐stimulation of hiPSC‐CMs for contractility and force maturation	hiPSC‐derived CMs	Pulse 5 V cm^−1^ 2 Hz frequency 5 ms pulse duration	Static stretch 4% stretch	‐	Enhanced RYR and SERCA expression	Improved contraction force Cell dimensions growth Increased tensile stiffness Maturation of excitation‐contraction coupling	2 weeks	[22]
Electroactive scaffold that provides EM‐stimulation in hiPSC	hiPSC	Biphasic stimulation 0.2 to −1 V 0.05,0.1,0.2 Hz	Uni and equiaxial M‐stimulation Cyclical stress of 17.5 Pa 0.3 Hz frequency	‐	Overexpression of cardiac markers: actinin, NKX2.5, GATA4, Myh6, c‐kit	Excellent cell viability hiPSC differentiation via EMAF scaffold	1 week	[130]

## 4 Engineering Adult‐Like CMs Using CPs

An ideal platform for engineering adult‐like CMs encompasses several key properties crucial for mimicking the native cardiac microenvironment. Biomimicry is fundamental, requiring the emulation of extracellular matrix architecture, cell‐cell interactions, and relevant biochemical cues to support the maturation of CMs. The substrate should possess topographical and structural features that guide the organization of CMs in alignment with the natural orientation observed in mature cardiac tissue. Incorporating materials with electrical conductivity within the conductivity range of the native myocardium (in the range of 5 × 10^−5^ to 1.6 × 10^−3^ S cm^−1^)^[^ ^133^ ^]^ is essential to facilitate electrical signaling and the maturation process. Mechanical properties mirroring the stiffness of native myocardium (of about 10 kPa to 1 MPa)^[^ ^134^ ^]^ are vital for providing cues for appropriate force generation and response. The inclusion of biochemical factors, signaling pathways, and support for interactions between different cell types within the cardiac microenvironment contribute to the platform's effectiveness. Tunability and customization are essential to accommodate variations in experimental design, cell types, and specific research goals. Furthermore, the platform should prioritize biocompatibility, scalability for large‐scale production, easy handling, and reproducibility to ensure consistent and reliable results across experiments and different laboratories. These properties collectively drive the success of strategies aimed at engineering mature and functional CMs, with CPs playing a role in fulfilling the criteria for an optimal cardiac tissue engineering platform.

### 4.1 Electrically Conducting Materials

Electrically conductive materials hold significance in regenerative medicine, especially within cardiac tissue engineering, where scaffolds require biocompatibility, electrical conductivity, mechanical stability, and architectural resemblance to the native myocardium.^[^ ^12^, ^135^, ^136^ ^]^ Effective propagation of electrical impulses within scaffolds is crucial to achieving synchronized contraction, a key aspect of cardiac function. CPs, in particular, have emerged as a promising tissue engineering tool due to their biocompatibility, electrical and ionic properties, flexibility, and versatility. CPs have demonstrated the ability to induce cellular mechanisms and appropriate responses to electrical fields in various tissues, including muscle, connective tissue, epithelium, and nervous tissue.^[^ ^29^ ^]^ CPs, often referred to as organic semiconductors, exhibit tunable properties akin to traditional metallic semiconductors, enabling them to mimic electrical behavior. Furthermore, their inherent softness affords enhanced mechanical adaptability within cells and organs, surpassing the capabilities of conventional inorganic electronic and metal materials. In the context of cardiac tissue engineering, CPs offer a unique avenue for creating scaffolds that not only support cell growth but also provide the necessary electrical cues for the maturation of CMs, ultimately advancing the field toward more effective regenerative strategies.

CPs exhibit inherent electrical conductivity owing to their chemical structure, consisting of alternating single (σ) and double (π) bonds. The π‐conjugated structure allows a certain extent of electron delocalization across the molecular backbone, thus enabling electron transfer.^[^ ^137^ ^]^ Typically, the electrical conduction within CPs arises from the facilitated electron jumps between molecular chains. These transitions are facilitated by the presence of dopant agents, which induce alterations in the electron density. This process involves the transfer of a charge from dopant molecules to polymer chains by means of charge carriers.^[^ ^138^, ^139^ ^]^ The effectiveness of the doping process and the characteristics of the resultant CPs are contingent upon a variety of factors, including the length of the polymer chain and its conjugation, as well as the efficiency of charge transfer, the type of dopants, and its molecular weight. CPs can undergo doping with both p‐type (positive charge) and n‐type (negative charge) dopants utilizing a diverse range of molecules. These include small ions like chloride (Cl^−^), bromide (Br^−^), or nitrate (NO_3_), as well as larger molecule dopants like hyaluronic acid, peptides, or polymers (PSS^−^).^[^ ^140^, ^141^ ^]^

The physicochemical characteristics of CPs greatly rely on the conditions under which they are synthesized. To ensure effective charge transport, it is imperative to maintain the conjugated structure of the monomer throughout the polymerization process. The synthesis of CPs can be accomplished through multiple approaches. Nevertheless, the oxidative coupling technique, involving monomer oxidation through either chemical or electrochemical means, stands out as the most commonly employed method. CPs can be fabricated through either chemical or electrochemical processes.^[^ ^142^, ^143^ ^]^

In chemical synthesis, CPs are typically synthesized through oxidative polymerization techniques. This involves the use of chemical oxidizing reagents, for example, iron(III) chloride or ammonium persulfate, to initiate the polymerization process, leading to the formation of highly conjugated polymer chains with extended π‐electron systems.^[^ ^144^, ^145^ ^]^ On the other hand, electrochemical routes leverage the application of an electric potential (or current) to induce the polymerization of monomers.^[^ ^146^ ^]^ Electrochemical polymerization provides good control over the polymer structure and allows for the deposition of the polymer directly onto electrodes. Both chemical and electrochemical methods offer unique advantages.^[^ ^147^ ^]^ More recent functionalized CP derivatives that have emerged as the dominant materials for sensing applications can only be synthesized using chemical methods.^[^ ^140^ ^]^ Chemical synthesis provides a variety of options to modify the CP backbone with more control over their physical and chemical properties and makes post‐synthesis covalent modification possible, which is particularly interesting in tissue engineering applications, along with the flexibility of matching the physical and chemical properties of native tissue. The main advantage of electrochemical synthesis is its ability to generate the material directly onto an electrode, which facilitates subsequent analysis and mitigates issues related to processability. Additionally, the electrochemical approach offers the benefit of precise control over polymer thickness, morphology, and degree of doping by adjusting synthesis parameters, such as the amount of charge introduced, type of doping ions, or electrolyte during the deposition procedure. However, electrochemical synthesis is limited to common conducting polymers in which the monomer can be directly oxidized upon application of potential (or current) at the electrode surface.

To gain a more extensive perspective of CPs we will discuss three of the most widely used CPs: poly(3,4‐ethylenedioxythiophene) (PEDOT), PPy, and polyaniline (PAni). PEDOT is a polythiophene‐based biomaterial, synthesized by the oxidative polymerization of the monomer 3,4‐ethylenedioxythiophene. Its applications range from tissue interfacing agents as it holds increased chemical and thermal stability,^[^ ^148^, ^149^, ^150^, ^151^ ^]^ to use for ionic and electrical conductivity.^[^ ^152^, ^153^ ^]^ PEDOT has been shown to possess a high cytocompatibility with different cell types, including CMs, stem cells, neurons, and fibroblasts.^[^ ^154^ ^]^ PPy is a well‐known CP with relevant features such as biocompatibility, simple synthesis, conductivity, and environmental stability.^[^ ^140^, ^155^, ^156^ ^]^ This material has stimulus‐responsive properties as well as in vitro and in vivo biocompatibility.^[^ ^157^ ^]^ Such properties give PPy the potential to control cellular activities such as DNA, cell migration, and proliferation.^[^ ^158^, ^159^ ^]^ A study showed improved CM function, overexpression of (α‐actinin, troponin T, and connexin 43), and electrical conductivity between 0.01 and 0.37 mS cm^−1^ with the use of electrospun PPy/PCL gelatin nanofibers.^[^ ^160^ ^]^ PAni has proven leading properties from the ease of synthesis to excellent electrical conductivity, environmental stability, and tunability of electrical cues by protonation or charge transfer doping.^[^ ^29^, ^161^ ^]^ PAni applications comprise electrically active redox polymers, pH‐switching electrically conductive biomaterials, and matrixes for nanocomposite CP.^[^ ^151^, ^162^, ^163^ ^]^ The oxidative polymeric product of aniline named PAni has been integrated as PAni‐PLGA aligned fibers to develop a 3D environment for the synchronous beating of CMs resulting in high expression of troponin T and gap junction protein connexin 43, increased electrical conductivity and improved cell adhesion.^[^ ^164^ ^]^

Other conductive elements such as metallic or semiconductor particles, carbon‐based nanomaterials, and piezoelectric materials are being used intensively for improving stem cell‐derived CM maturation.^[^ ^165^ ^]^ Each of them presents a range of advantages and disadvantages when compared and contrasted to CPs.

Gold particles are biocompatible structures that reinforce and enhance the electrical conductivity of engineered constructs.^[^ ^166^ ^]^ Dvire et al. incorporated gold nanowires into a macroporous alginate scaffold connecting the non‐conducting pore walls and facilitating adjacent CM communication which revealed high expression of α‐sarcomeric actinin, troponin I, and connexin 43.^[^ ^167^ ^]^ Although gold‐based particles offer better biocompatibility than some CPs, they can interfere with the scaffold crosslinking and can dissociate from the scaffold once implanted in vivo.^[^ ^168^, ^169^ ^]^

MXenes are a family of 2D transitional metal carbides and nitrides derived from the “MAX phases” layered ceramics. 2D MXene (Ti_3_C_2_T*_x_*) was covalently crosslinked with collagen type I as a conductive platform to mature hiPSC‐CMs cultured under an electric field stimulation.^[^ ^170^ ^]^ This biohybrid system resulted in the improvement of cell proliferation and significant expression of connexin 43 while limiting bacterial attachment and proliferation. Although MXenes possess antimicrobial properties which CPs lack, as well as high hydrophilicity and electrical conductivity, they are limited by their mechanical brittleness and ambient oxidation.^[^ ^171^, ^172^, ^173^ ^]^

Carbon nanomaterials can be incorporated into soft hydrogels to tune their mechanical and electrical environments and make them physiologically‐mimicking.^[^ ^174^ ^]^ Dispersion of multiwall CNTs in pericardial matrix hydrogel was shown to trigger the proliferation of CMs and increase cardiac‐specific gap junction protein (connexin 43).^[^ ^175^, ^176^ ^]^ Graphene is a crystalline allotrope of carbon consisting of a single layer of carbon atoms obtained from chemical and physical modifications.^[^ ^177^ ^]^ Wang et al. successfully differentiated hiPSCs on graphene sheets into functional CMs that, in the absence of E‐stimulation, showed an increased conduction velocity and myofibril ultrastructural organization with enhanced electrophysiological properties.^[^ ^178^ ^]^ Graphene oxide and reduced graphene oxide, derivatives of graphene are usually preferred for their more stable and tunable properties. These derivatives also favor cell adhesion. Carbon‐based nanomaterials have a higher surface area than CPs which allows them to load and release large amounts of bioactive molecules, but, they suffer from a particle size and dose‐related long‐term toxicity. Moreover, their high Young's modulus can greatly increase the stiffness of scaffolds, which can adversely affect cell behavior. In addition, when incorporated into polymeric systems, they suffer from a limited electrical conductivity and a lack of control over their dispersion.^[^ ^177^ ^]^

### 4.2 CPs Fiber‐Like Scaffolds

In tissue engineering, it is well‐known that the scaffold architecture has a very strong impact on cellular behavior.^[^ ^179^ ^]^ The topography is mainly imposed by the morphology of the scaffold on which the CP is deposited. Numerous techniques have been developed to produce scaffolds whose properties must mimic those of the natural extracellular matrix (ECM) with its fibrous architecture with high porosity and pore size. Indeed, the morphological, mechanical, and biochemical cues are known to play a key role in cardiac cell behavior and maturation.^[^ ^180^ ^]^ A combination of micro and nanoscale topographies on the surface of the scaffolds also strongly favors the maturation of CMs.^[^ ^181^ ^]^

Various materials and techniques have been used to fabricate scaffolds with different properties.^[^ ^182^ ^]^ Natural or synthetic polymers can be employed but often, a blend of them is preferred to improve the overall properties of the scaffold. Natural polymers like collagen, alginate, silk fibroin, gelatin, elastin, etc. have the advantage of promoting cell attachment and proliferation but some additional chemical steps may be needed to improve their solubility or tune their biodegradability.^[^ ^183^ ^]^ Synthetic biodegradable polymers such as poly(lactic acid) (PLA), poly(glycolic acid) (PGA), polycaprolactone (PCL), polyvinyl alcohol (PVA), polyethylene glycol (PEG), polyurethane (PU), etc. have easily tunable mechanical properties but their Young's modulus usually remains too high to obtain very soft and flexible scaffolds. To improve both their mechanical and biochemical properties, blends of synthetic and natural polymers are often considered.^[^ ^184^ ^]^ An alternative is to use specific scaffold designs but these approaches are more complex to implement.^[^ ^185^ ^]^

2D and 3D scaffolds can be fabricated using different techniques such as 3D printing, electrospinning, freeze casting, self‐assembly, etc.^[^ ^186^ ^]^ Among them, electrospinning is often the preferred approach to obtain electrospun scaffolds with aligned micro and nanofibers. These aligned scaffolds, produced with high‐speed spinning drums or rotating collectors, feature a high porosity level with a heterogeneous distribution of pore size which recapitulates the main structural characteristics of the ECM.^[^ ^180^ ^]^ Once these scaffolds are fabricated, the next step consists of depositing the CP by direct immersion of the scaffolds in a solution containing the CPs, by vapor‐ or liquid‐phase polymerization, or by electrodeposition.^[^ ^187^ ^]^

A critical issue concerns the elastic properties of the scaffold that will depend on Young's modulus of the electrospun material and that of the deposited CP.^[^ ^180^, ^188^ ^]^ Very soft electrospun polymers must be used with Young's modulus of about tens to hundreds of kPa because the subsequent deposition of the CPs onto the scaffold will increase its stiffness as its Young's modulus is about a few tens of MPa.^[^ ^189^ ^]^ A compromise must therefore be found in terms of the deposited layer thickness to limit the increase in stiffness while ensuring an efficient electromechanical actuation. The latter is also strongly dependent on the porosity of the deposited CP layer. A dense layer will generate a high strain at low frequencies while a porous one will lead to a smaller strain at higher frequencies.

It is also possible to obtain conductive electrospun scaffolds using electrospinning of CPs or co‐electrospinning of blends of CPs and other polymers.^[^ ^186^, ^187^, ^190^ ^]^ In that case, the conductive scaffolds can be directly produced by electrospinning without the need for additional deposition steps to make it electrically conductive.

### 4.3 Intrinsic Electrical Conduction of CPs

As mentioned previously, a conductive scaffold can play a decisive role as a passive element responding to electrical signals generated by the CMs or as an active element with which E‐stimulation can be applied to the CMs. Due to electrical signal transmission through the scaffold over long distances, it has been shown that electrically conductive scaffolds improve the synchronization of cardiac cell beating in the myocardium. Indeed, for CMs grown on conductive 2D and 3D scaffolds, excitation‐contraction is promoted and intercellular gap junctions are better created (connexin 43).^[^ ^191^, ^192^ ^]^ These properties are further enhanced if conductive scaffolds with aligned nanofibers are employed.^[^ ^193^ ^]^

Beneficial effects for CM maturation have been observed without applying any external E‐stimulation using a conductive biodegradable scaffold made of PCL blended with aniline pentamer^[^ ^194^ ^]^ or a biohybrid hydrogel composed of collagen, alginate, and PEDOT:PSS. Numerous studies have shown that the use of conductive scaffolds made of CPs is of particular relevance for designing cardiac patches.^[^ ^195^, ^196^, ^197^, ^198^, ^199^ ^]^

Indeed, the conductive properties of CPs are intrinsically linked to the use of dopants. Their ionic properties give rise to an inherent charge that can favor cell adhesion through electrostatic forces as evidenced by B1‐integrin and integrin‐linked kinase upregulation.^[^ ^181^ ^]^ Adsorption of proteins and control of their conformation on electrically charged scaffolds are also improved which might increase biochemical cues for the maturation of CMs.^[^ ^200^, ^201^ ^]^ Moreover, the contact of the cell membrane with soft conductive CPs facilitates the reinforcement of an electrical coupling that triggers specific phenotype switching that is known to promote CM maturation.^[^ ^193^ ^]^

### 4.4 E‐Stimulation through CPs

**Figure 6:**
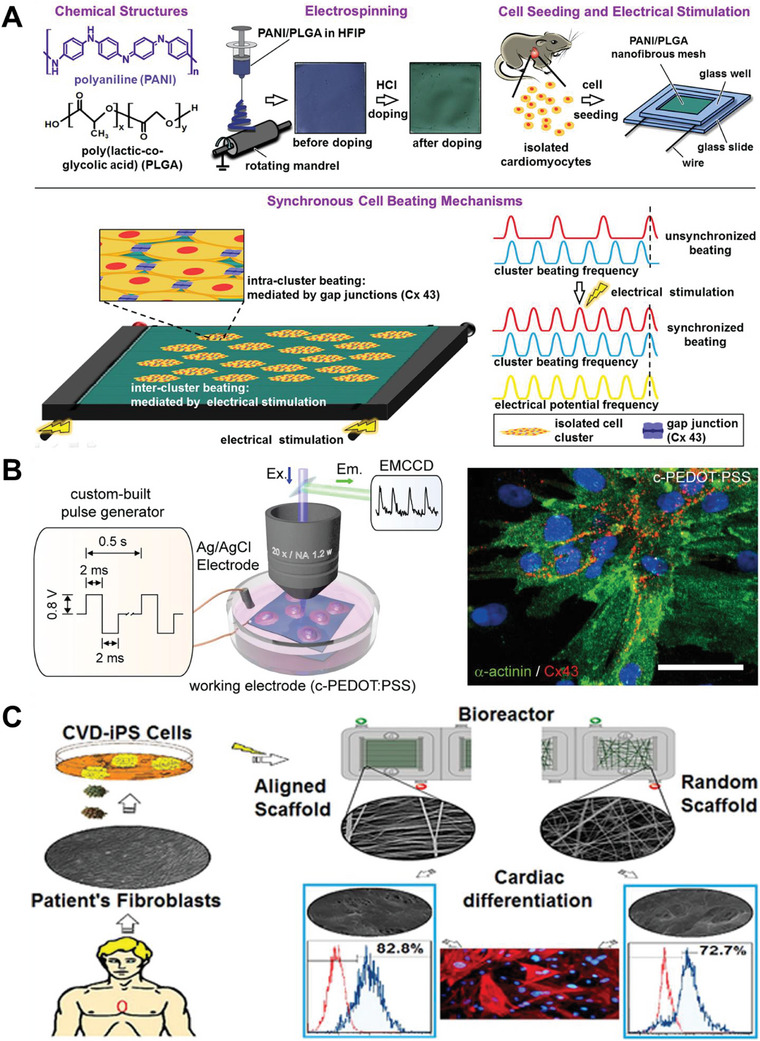
A) Schematic diagrams demonstrating the synchronization of cell beatings following E‐stimulation of cells seeded on aligned conductive nanofibrous mesh. Reproduced with permission.^[^ ^191^ ^]^ Copyright 2013, Elsevier. B) A schematic diagram and an immunofluorescence image showing the direct E‐stimulation and optical imaging of neonatal CMs stained for DAPI (blue), α‐Actinin (green), and connexin 43 (red) with a scale bar of 100 µm. Reproduced with permission.^[^ ^204^ ^]^ Copyright 2018, Springer Nature. C) Schematic representation of the E‐stimulation of CVD‐specific hiPSCs in a unidirectional and multidirectional manner. Reproduced with permission.^[^ ^205^ ^]^ Copyright 2017, American Chemical Society.

E‐stimulation using CPs has emerged as a promising approach to promote the development of sarcomeres and enhance the synchronized, rhythmic contraction of cardiac cells.^[^ ^202^ ^]^ The use of conductive composite substrates, both in 2D and 3D forms, has been demonstrated to accelerate maturation and enhance the contractile and electrical characteristics of CMs. The scaffold consists of a non‐conductive component that creates a biocompatible microenvironment for cultured CM cells. Meanwhile, the CP component enhances intercellular coupling and facilitates electrical signal propagation. Kai and colleagues successfully synthesized PPy‐contained nanofibrous membranes composed of polycaprolactone (PCL) and gelatin.^[^ ^203^ ^]^ The conductive scaffolds were evaluated, and those containing 15% PPy demonstrated a harmonious blend of conductivity, mechanical strength, and degradability, making them suitable for cardiac tissue applications. These conductive nanofibers supported primary CM attachment, proliferation, and differentiation effectively. However, the E‐stimulation of CMs through the conductive nanofibrous scaffolds was not demonstrated. In another investigation by Hiao et al., CMs were cultured on aligned nanofibers made of PANi and PLGA, as an electrically active scaffold for coordinating the beatings of the cultured cells.^[^ ^191^ ^]^ After undergoing a doping process through HCl treatment, the electrospun nanofibers could be converted into a conductive state with positive charges. Consequently, the fibers exhibited a better ability to attract negatively charged adhesive proteins, such as fibronectin and laminin, resulting in enhanced cell adhesion. The CMs expressed gap‐junction proteins (connexin 43) and exhibited synchronous beating within individual cell clusters, signifying the establishment of electrical coupling among cells. The beating frequencies could be controlled by external E‐stimulation using the CP as shown by Hsiao et al. (**Figure** **6**A). These results suggest the electrical field generated on the conductive scaffold plays a significant role in coordinating the contraction between the isolated cell clusters. Similarly, Kim and colleagues developed direct cellular interfaces based on densified nanofibrillar PEDOT:PSS via a solvent‐assisted crystallization process on a polyethylene terephthalate (PET) substrate.^[^ ^204^ ^]^ The PEDOT:PSS exhibited excellent electrical characteristics, long‐term underwater stability without film dissolution, and good viability of primarily cultured CMs. The crystalized nanofibrillar conducting polymer networks enabled enlarged surface areas, which were successfully employed to modulate CM beating by applying electrical pulses to modulate their synchronized beating frequencies (Figure 6B). Amirabad et al. demonstrated that unidirectional electrical pulses for 1 h d^−1^ for 15 days can enhance the cardiac differentiation of CVD‐specific hiPSCs (Figure 6C).^[^ ^205^ ^]^

### 4.5 Unique EM‐Stimulation through CPs

**Figure 7:**
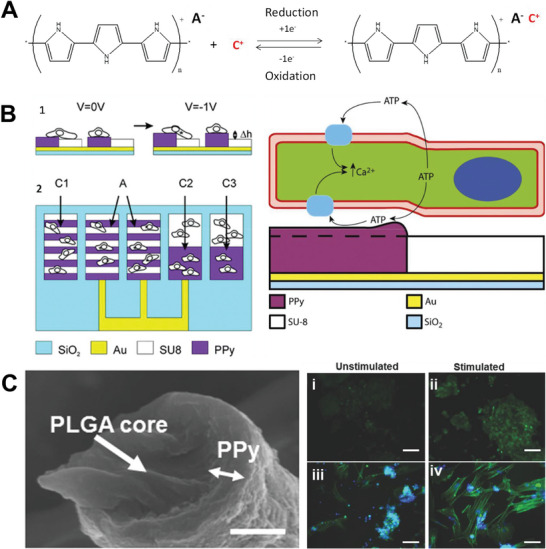
A) Schematic representation of the chemical mechanism responsible for PPy actuation. B) Schematic representation of the design and fabrication of an M‐stimulation chip using PPy microactuators resulting in a cellular Ca^2+^ response induced by autocrine ATP signaling. Reproduced with permission.^[^ ^216^ ^]^ Copyright 2011, The Royal Chemical Society. C) SEM image of the cross‐sectional view of the PLGA core coated with PPy (scale bar = 500 nm) and microscopic images of iPSCs cultured electromechanically stimulated and unstimulated scaffolds stained for i,ii) live/dead and iii,iv) actin filaments. Reproduced with permission.^[^ ^130^ ^]^ Copyright 2016, Wiley‐VCH GmbH.

CPs are also a class of ionic electromechanical active materials widely investigated for the development of soft, high‐stress, large‐strain, and low‐driving voltage actuators. The actuation mechanism of CPs resembles that of natural muscles in terms of being wet, soft, and responsive to external E‐stimulation and control. By applying a low potential (typically below 1 V), CPs undergo a change in volume through electrochemical oxidation and reduction reactions.^[^ ^206^ ^]^ This volume alteration primarily occurs due to the movement of ions and solvents within the polymer matrix during electrochemical redox cycling. **Figure** **7**A illustrates the chemical mechanism for PPy doped with large, immobile anions (A^−^), for example, PSS^−^, DBS^−^ in an electrolytic media containing both mobile cations (C^+^) and anions. As PPy is reduced, cations C^+^ are inserted and reversely expelled when the polymer is oxidized to compensate for the charge imbalance. More simply, the polymer expands in its reduced state, and contracts when a positive potential is applied (oxidize state). This reversible process generates significant stress ranging from 1–5 MPa and a strain of a few percent (1–15%).^[^ ^207^, ^208^ ^]^ The actuators can operate in virtually any aqueous salt solution, blood plasma, urine, or cell culture medium.^[^ ^209^ ^]^

Only a limited number of CPs have been utilized for the design of electromechanical active materials. The primary focus for material selection revolves around the use of PPy, which offers distinct advantages, including the ability to generate significant actuation strain and stress, as well as the ease of production using either aqueous solutions or organic electrolytes.^[^ ^210^, ^211^, ^212^, ^213^ ^]^ However, PPy also presents various limitations, such as low electrical conductivity, slow ion diffusion rate, and the risk of over‐oxidation.^[^ ^214^ ^]^ Here, PEDOT has been the subject of extensive investigations due to its remarkable electrical conductivity exhibits conductivity that is one order of magnitude higher than that of PPy, along with excellent biostability and biocompatibility.^[^ ^215^ ^]^ Meanwhile, the synthesis of CPs is simple and controllable either by oxidative chemical or electrochemical methods, electrochemical synthesis has emerged as the preferred method for actuator applications due to its simplicity, high selectivity, and reproducibility.^[^ ^144^ ^]^ This approach offers several advantages: It allows the CP material to be directly synthesized on a conductive electrode, simplifying subsequent analysis and eliminating challenges related to processability. Additionally, the electrochemical route enables precise control over key polymer characteristics, including thickness, morphology, electrical conductivity, and degree of polymer doping, by effectively managing synthesis parameters such as the injected charge quantity during the deposition process.

The remarkable redox deformability of CPs, in addition to their excellent biocompatibility, make them promising for the development of electromechanically active tools that can safely interact with living systems. Studies conducted by Jager and colleagues showcased the production of PPy actuator chips designed for mechanically stimulating individual renal epithelial cells.^[^ ^216^, ^217^ ^]^ These chips were manufactured using conventional microfabrication techniques on silicon substrates. The active component of the chip consisted of the bulk CP PPy, which exhibited expansion upon the application of a low potential (1 V) (Figure 7B). The cells demonstrated favorable adhesion and spreading on the chip's surface. The PPy effectively stretched the individual cells, and the cellular responses were captured through live fluorescent imaging, revealing an increase in intracellular Ca^2+^ levels. This Ca^2+^ response was attributed to an autocrine ATP signaling pathway associated with the M‐stimulation of the cells.

However, the efficient electromechanical actuation of CPs often faces challenges such as their high Young's modulus (≈100–200 MPa) and low elasticity caused by their brittle structure. To address these issues, the CP aimed at cellular M‐stimulation needs specific properties. These include elasticity, a low Young's modulus to prevent efficiency loss of the electrically driven volume change in the actuating polymer, and strong adhesion of the deposited CP to prevent delamination and creep during electromechanical actuation. To address these challenges, various approaches have been explored to enhance the mechanical properties of CPs. One method involves incorporating appropriate dopants or incorporating CPs into cross‐linked elastic scaffold structures. For example, a study conducted by Gelmi et al. demonstrated that a fibrous scaffold made of PLGA and coated with PPy was able to deliver both E‐ and M‐stimulations to CMs derived from (hiPSCs).^[^ ^218^ ^]^ This electroactive scaffold demonstrated a higher expression of cardiac markers in the stimulated protocols compared to the unstimulated ones, indicating its effectiveness in promoting cardiac regeneration (Figure 7C). In another similar study, E. Kerr‐Phillips et al. developed an electromechanical active elastomeric scaffold that shows controllable pore size variation upon oxidation and reduction of the conducting polymer. The electrospun nanofibrous mats consist of a cross‐linked nitrile butadiene rubber combined with the CP PEDOT.^[^ ^219^ ^]^ While no experiments were conducted with living cells, these electroactive mats hold promise as potential candidates for developing 3D electroactive scaffolds for studying mechanical transduction in CM cells.

CPs are being used more frequently in biomedical systems; however, CM maturation approaches hold several challenges to be addressed. First, it is very likely that CMs receive heterogeneous E‐stimulation within the scaffold.^[^ ^35^ ^]^ This limitation will need to be tackled in the future to prevent in vitro scaffolds from having CMs fully matured portions and partially matured portions. Second, electrical conductivity varies widely between polymers, and their applications are limited to distinct tissues so there is a long path in CP discovery. CPs hold a potential use in biomedical applications and clinical needs. Prospects include the use of CPs for the treatment of lagophthalmos, nerve regeneration stimulation, myocardial regeneration, electrically induced targeted drug release systems, and deep brain stimulation treatments.^[^ ^29^ ^]^

### 4.6 Use of CPs for In Vivo Applications

The use of CPs is not limited to in vitro applications. It has been introduced for in vivo applications as well, specifically as a therapeutic strategy to treat myocardial infarction (MI). During an MI the damaged area of the heart develops scar tissue, increases resistivity, and experiences dys‐synchronous contraction. CPs represent a novel platform to selectively restore the electrical conductivity in infarcted heart tissues. For cardiac tissue regeneration, two main strategies can be distinguished: the use of sutured or glued electroconductive cardiac patches or the use of injectable conductive hydrogels.^[^ ^220^ ^]^ It is also crucial that both strategies allow for the controlled release of active substances like growth factors, genes, etc.

Miharda and colleagues developed an alginate‐PPy‐based injectable hydrogel to treat a rat‐infarcted myocardium achieving overall conductivity of 10^−2^ S cm^−1^, angiogenesis after 5 weeks post‐injection and recruitment of myofibroblasts into the infarct area.^[^ ^221^ ^]^ In a different study, conductive chitosan/PPy hydrogels were used to treat induced MI rat hearts, improving the ejection fraction and fraction shortening after 3 months post‐treatment.^[^ ^222^ ^]^ Multipolymer formulations offer a variety of therapeutics properties and this was the case in a PPy, highly branched poly(β‐amino ester), gelatin hydrogel showing a therapeutic 4 weeks after injection including an improved ejection fraction/fractional shortening to 56%/31%, end diastolic volume/end systolic volume to 525/420 µL and prolonged QRS of 40 ms.^[^ ^223^ ^]^ In addition to combinatorial polymeric approaches, cell encapsulated patches show promising effects in vivo, a CM‐laden/poly‐3‐amino‐4 methoxybenzoic acid/gelatin (PAMB‐G) patch injected to MI rat hearts achieved: 1) reduced scar‐related resistivity as observed on a 3.4‐fold increase in field potential 2) electric pulse propagation two times faster than the controls 3) reduced scar tissue size and 4) shorter QRS intervals.^[^ ^133^ ^]^

Despite the remarkable integration of CPs into in vivo research work, there are three fundamental challenges to fully address translation to in vivo; 1) Investigation of the therapeutic effects from cells and CPs independently to precisely integrate the most suitable cell types and polymers 2) Standardization of the parameters to track therapeutic effect in situ and not as a result of the complete heart mechanics. With the variety of MI models and physiological metrics measured by different groups, it becomes unclear which strategies reveal the most reliable effects and how they compare to each other. 3) Measurement of the long‐term versatility and performance of the CPs in terms of cardiovascular functional biomechanics and electrical conductivity.^[^ ^192^ ^]^

## 5 Conclusion

The quest for mature CMs derived from hiPSCs represents a critical step toward advancing our understanding of cardiac diseases and developing effective therapeutic interventions. Despite significant progress in obtaining hiPSC‐derived CMs with high purity and yield,^[^ ^14^, ^224^, ^225^ ^]^ their immature phenotype remains a limitation in translational applications, particularly in drug discovery and regenerative medicine.

The importance of developing reproducible, scalable, and high‐throughput manufacturing strategies to generate adult‐like CMs cannot be overstated. Adult‐like CMs are crucial for accurately modeling cardiac diseases that predominantly manifest in adulthood. These mature cell populations hold great potential for disease modeling, drug screening, and personalized medicine, allowing for more effective and targeted therapies.

However, the road to achieving adult‐like CMs is riddled with challenges. One of the primary limitations lies in the current strategies employed for CM maturation. While efforts have been made to replicate the native cardiac environment using scaffolds and biomaterials, the implemented systems often fall short of fully capturing the dynamic and complex interactions that occur within the extracellular matrix of the heart. Fine‐tuning the composition, architecture, and elasticity of the scaffolds becomes critical to provide an environment that supports the maturation process.

To enhance the maturation of CMs, the integration of biochemical, electrical, and mechanical stimuli is indispensable. These stimuli are essential for replicating the natural cues that drive CM development and functionality. Advanced devices, such as electro‐mechano‐active polymer‐based scaffolds (EMAPS) based on CPs, offer a promising approach. EMAPS can provide topographical, biochemical, and electromechanical stimuli to the cells, closely mimicking the physiological conditions and promoting their maturation.

While EMAPS shows potential, critical considerations must be addressed. One critical point is the lack of a 3D system that mimics the natural environment of the cells. While open systems allow for easier exchange of nutrients, oxygen, and biochemical cues, they do not fully replicate the complex interactions that occur in the native cardiac environment. The trade‐off between 3D microenvironments and the exchange of nutrients and biochemical cues must be carefully evaluated. Biocompatibility and biodegradability of the materials are crucial considerations to ensure the long‐term viability and integration of CMs. The use of degradable conducting polymers comprising electroactive oligomer units that form degradable ester linkages might pose a solution for this issue.^[^ ^226^, ^227^ ^]^ The adhesion of cells to the scaffold surface is also important for their proper functionality. Design considerations and electrical connections are important aspects that must be considered to achieve optimal functionality and integration within such systems. Biochemical cues can be loaded and released by EMAPS, however, it requires a great deal of optimization to adapt their delivery rate through E‐stimulation as the CMs mature. Additionally, the integration of EMAPS into more sophisticated systems, with precise control over topographical, biochemical, and electromechanical stimuli, is necessary for achieving a high degree of CM maturation.

In conclusion, the journey toward obtaining mature and functional CMs from hiPSCs is an ongoing endeavor that requires the development of reproducible manufacturing strategies and innovative scaffold designs. Integration of EMAPS, with their ability to provide all the required stimuli, holds promise in advancing the field. However, critical points such as biocompatibility, degradation, cell adhesion, longevity, and system integration must be thoroughly addressed to enhance the maturation and functionality of hiPSC‐CMs. These advancements will not only enhance our understanding of cardiac diseases but also facilitate the discovery of novel therapeutics and personalized treatment strategies for patients with cardiovascular disorders.

## Conflict of Interest

The authors declare no conflict of interest.
